# The Comparison of Adipose Stem Cell and Placental Stem Cell in Secretion Characteristics and in Facial Antiaging

**DOI:** 10.1155/2016/7315830

**Published:** 2016-02-08

**Authors:** Yan Xu, Shilei Guo, Cui Wei, Honglan Li, Lei Chen, Chang Yin, Chuansen Zhang

**Affiliations:** ^1^Nanjing Regenerative Medicine Engineering and Technology Research Center, Nanjing 210046, China; ^2^Regenerative Medicine Center, The Second Military Medical University, Shanghai 200433, China

## Abstract

*Background.* Mesenchymal stem cells are the most commonly used seed cells in biomedical research and tissue engineering. Their secretory proteins have also been proven to play an important role in tissue healing.* Methods.* We isolated adipose stem cells and placental stem cells and performed analysis examining characteristics. The secretory proteins were extracted from conditioned medium and analyzed by MALDI-TOF/TOF. The antiaging effect of conditioned mediums was evaluated by the results of facial skin application.* Results.* Adipose stem cells and placental stem cells were found to be very similar in their surface markers and multipotency. The specific proteins secreted from adipose stem cells were more adept at cell adhesion, migration, wound healing, and tissue remodeling, while the proteins secreted by placental stem cells were more adept at angiogenesis, cell proliferation, differentiation, cell survival, immunomodulation, and collagen degradation. While these two types of conditioned medium could improve the facial index, the improvement of Melanin index after injection of the adipose stem cell conditioned medium was much more significant.* Conclusion.* The results suggest that the secreted proteins are ideal cell-free substances for regeneration medicine, especially in the antiaging field.

## 1. Introduction

Mesenchymal stem cells are a type of adult stem cell with the attributes of self-renewing and multipotency. They can be used in biomedical research, new drug development, and toxicology studies, and so forth [1]. Currently this type of cell is a focal point in clinical research and application [2–4].

The phenomenon that mesenchymal stem cells could improve distal injury organ healing without cell migration and cell differentiation indicates that the cell exosomes play an important role in tissue and organ repair [5–7]. In addition to cell differentiation and proliferation, the secreted proteins also represent an important extension of the stem cells' function [8, 9].

In recent years, adipose stem cells (ASCs) have received great attention in regenerative medicine. The research shows that ASCs are both abundant and varying in activities within our bodies [10]. The ASCs also have very similar characteristics and classification with bone marrow mesenchymal stem cells. Therefore, the proliferation, differentiation, and secretome characteristics of ASCs are considered advantageous in tissue protection, antiapoptosis, and cell replacement [11]. In addition, fat tissue is easily harvested in plastic surgery, making ASC an accessible and ideal kind of adult stem cell.

ASCs have the problem of being limited in cosmetics and clinical application. Due to the requirement of having a large quantity for clinical application, we also chose to use placental stem cells (PSCs) in this secretomics research [12]. Placental tissue is a crucial accessory in protection, nutrition, respiration, and excretion of the embryo. Researchers isolated mesenchymal stem cells from mature placenta and found that PSCs express the typical genes of mesenchymal stem cells as well as some other specific gene [13]. In addition, the placenta is an important nutrient for the development of the fetus and can secrete many developmental factors, such as granulocyte colony-stimulating factor (G-CSF), granulocyte-macrophage colony-stimulating factor (GM-CSF), macrophage colony-stimulating factor (M-CSF), and stem cell factor (SCF) [14]. From this, we speculate that PSCs have unique characteristic of their secretome.

The characterization of secretomics of mesenchymal stem cells was unclear because of the differences in culture condition, culture medium, expansion time, and so forth. In our research, we utilized a method for the extraction of secretory proteins suitable for clinical application and analysis of the secretomes of ASCs and PSCs. Furthermore, we examined the antiaging ability of their secretion factors in human facial skin.

## 2. Methods

The human trials in this study obtained the informed consent of the volunteers according to the Helsinki Declaration and were approved by the Society of Medical Ethics in Jiangsu Province.

### 2.1. Isolation of Adipose Stem Cells and Placental Stem Cells

A single sample of 100 mL of adipose tissue was first harvested from a 45-year-old female cosmetic surgery patient. The adipose tissue was washed three times with sterile phosphate-buffered saline solution (PBS). The washed tissue was cut into pieces and digested by collagenase (type I collagenase, Worthington, USA) for forty minutes at 37°C with gentle agitation. Enzyme activity was neutralized with Dulbecco Modified Eagle Medium (DMEM) supplemented with 2% fetal bovine serum (FBS), and the tissues were filtered with 150 *μ*m filters. After centrifugation at 300 g for 10 minutes, the cell pellet was resuspended in DMEM containing 2% human platelet lysate (hPL) and 1% penicillin-streptomycin, cultured, and expanded at 37°C in 5% CO_2_ incubators.

The placental tissue was washed with PBS to remove the blood. The tissue was minced into small pieces and incubated with 0.2% collagenase II for one hour at 37°C to allow for thorough digestion and filtered through a 250 *μ*m metal sieve to remove the tissue fragments. The cells were collected by centrifugation at 300 g for 10 min and washed three times with PBS. The cell pellet was then suspended in DMEM with 2% hPL and 1% penicillin-streptomycin and incubated at 37°C in 5% CO_2_ incubators.

### 2.2. Characteristics of Adipose Stem Cells and Placental Stem Cells

To confirm multipotency, the differentiability of the cells into osteoblasts, adipocytes, and chondrocytes was analyzed. Both types of cells were plated at a density of 3 × 10^4^ cells/cm^2^ in dishes at passage 3. Once the cells were 90% confluent, the completed medium was substituted with inducing medium (osteogenic medium: completed medium supplemented with 50 *μ*M ascorbic acid, 0.1 *μ*M dexamethasone, and 10 mM b-glycerolphosphate; adipogenic medium: complete medium supplemented with 1.0 *μ*M dexamethasone, 10 mg/mL insulin, 100 *μ*M indomethacin, and 500 *μ*M IBMX; chondrogenic medium: completed medium with 1% penicillin-streptomycin, 1% ITS (insulin, transferrin, and selenium), 100 nM dexamethasone, 50 *μ*g/mL L-ascorbic acid 2-phosphate, 10 ng/mL TGF*β*1, and 500 ng/mL BMP6). For up to 20 days, the cells were stained with Oil Red O for adipogenic induction, Alizarin Red S for osteogenic induction, or Safranin O for chondrogenic induction. For fluorescence activated cell sorting (FACS) analysis, the cultured cells were collected and incubated with CD29-fluorescein isothiocyanate (FITC), CD34-FITC, CD71-FITC, and CD90-FITC for 30 min at 4°C and then analyzed using flow cytometry.

### 2.3. Extraction of Secretory Protein in Conditioned Medium (CM)

The ASCs and PSCs culture dishes were washed three times with PBS and cultured overnight in culture medium consisting of DMEM, Nutrient Mixture F-12 (DMEM/F-12), and 0.2% hPL resulting in about 6–8 × 10^6^ cells. After 24 hours, the cultured medium was collected and fresh serum-free medium was added. The CM was filtered using a 0.22 *μ*m filter, centrifuged with Amicon Ultra 15 mL (MWCO 3 kD, Millipore) at 4°C 4000 g for 30 minutes, and then concentrated about 5 times using ultrafiltration membrane of 3.5 kD with polyethylene glycol at 4°C. Overall, the CM was concentrated about 15 times in the above process. The initial concentration was 1000 mL of 0.389 ± 0.04 mg/mL and the final concentration was 70 mL of 5.989 ± 0.07 mg/mL. Freeze-dry powder was prepared in sterilized penicillin bottles using the Lyophilizer (Boyikang Corp., Beijing, China).

### 2.4. MALDI-TOF/TOF Analysis

Protein identification experiments were performed at positive mode using the MALDI-TOF/TOF 7090 system. In-gel tryptic digestion of the proteins was performed using an in-gel digestion kit following the protocol recommended by the manufacturer (Shimadzu Kratos, Kyoto). Before deposition onto a MALDI plate, all samples were desalted and concentrated with C18 ZipTip following the recommended protocol (Millipore, Massachusetts). Peptide extracts were eluted in a concentrated solution of 2,5-dihydroxybenzoic acid (12.5 mg/mL) with 50% acetonitrile and 0.1% trifluoroacetic acid in water and spotted onto the MALDI target plate. The TOF spectra were recorded in the reflector mode with a mass range from 500 to 2000 Da. Each spectrum was the cumulative average of 200 laser shots. The spectra were calibrated with the trypsin autodigestion ion peaks *m*/*z* (842.51 and 2211.10). The peptide mass fingerprints (PMF) were obtained using the Mascot search engine with a tolerance of 100 ppm and one missed cleavage site.

### 2.5. Application in Facial Antiaging

18 young volunteers were randomly divided into 3 groups with 6 in each group: ASC-CM group, PSC-CM group, and control group. The CM of ASC or PSC was dissolved into injectable hyaluronic acid (HA) and injected into the facial skin of each person with DermaQueen equipment (Seoul, Korea). The injected depth was 0.1 mm, the interval was about 2 mm, and the volume was 2 *μ*L in each point. The ASC-CM group was injected with HA containing 2 mg/mL ASC-CM, the PSC-CM group was injected with HA containing 2 mg/mL PSC-CM, and the control group was injected with HA containing 0.2% hPL. 15 days after injection, the facial skin of each volunteer was tested with Courage & Khazaka Electronic GmbH (Cologne, Germany). The detection indexes included Erythema, Melanin, Glossymeter, TEWAmeter, and Corneometer. The intensity of skin redness was measured using the Erythema index (Erythema). The Melanin index represents the darkness of the skin (Melanin). Glossymeter is used to measure gloss on skin. TEWAmeter is used to assess the transepidermal water loss (TEWL) and evaluated the water barrier function of the skin. Corneometer can determine the water content in skin.

### 2.6. Statistical Analysis

The measurement data was described using mean and standard deviation and the difference was analyzed using paired *t*-test and analysis of variance (ANOVA). All the analyses were performed by Stata (12.0 version) using *p* < 0.05 as statistically significant.

## 3. Results

### 3.1. Characterization of Adipose Stem Cells and Placental Stem Cells

**Figure 1:**
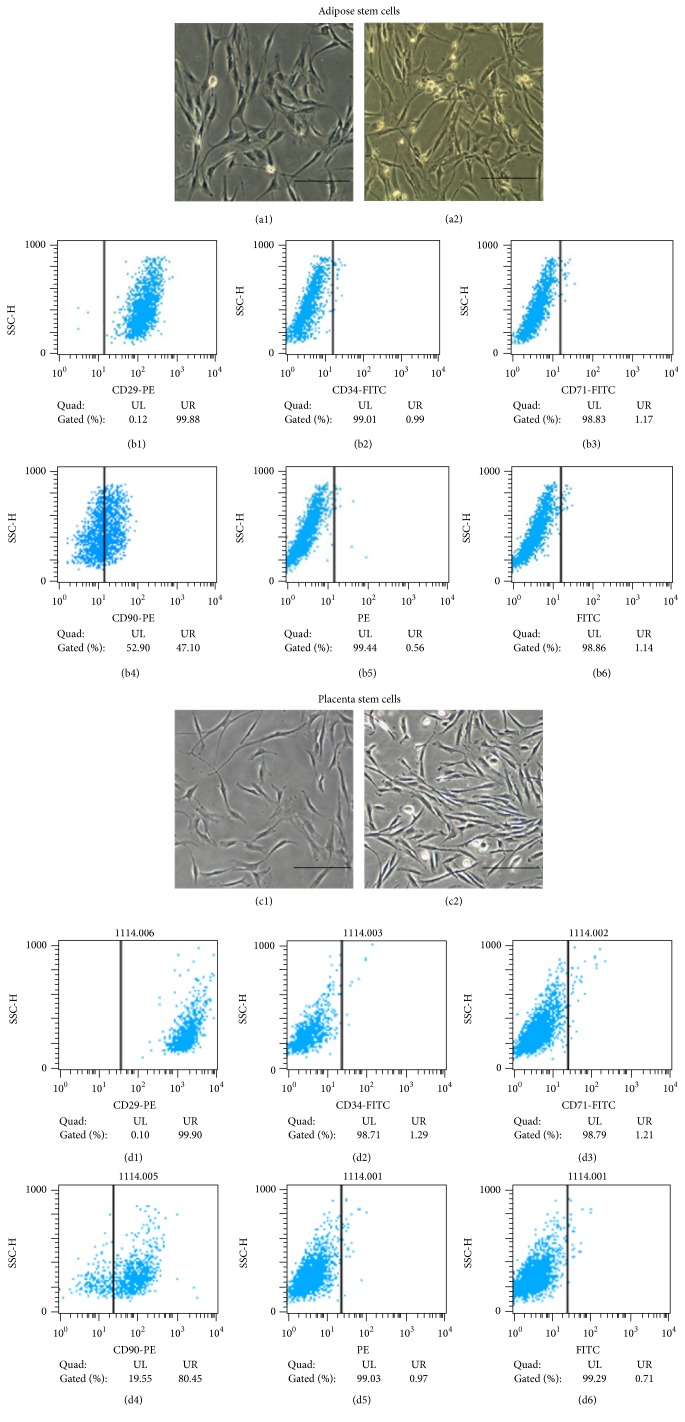
Characterization of adipose stem cells and placenta stem cells. Both cells showed typical fibroblast-like morphology at passage 1 (a1, c1) and passage 3 (a2, c2). The flow cytometry analysis confirmed that adipose stem cells express CD29 (99.88%, b1) and CD90 (52.9%, b4) but do not express CD34 (0.99%, b2) and CD71 (1.17%, b3); the placenta stem cells express CD29 (99.9%, d1) and CD90 (80.45%, d4) but do not express CD34 (1.29%, d2) and CD71 (1.21%, d3). The FITC (b6, d6) and PE (b5, d5) were performed as blank control. UL refers to upper left quadrant and UR refers to upper right quadrant. Scale bar = 100 *μ*m.

**Figure 2:**
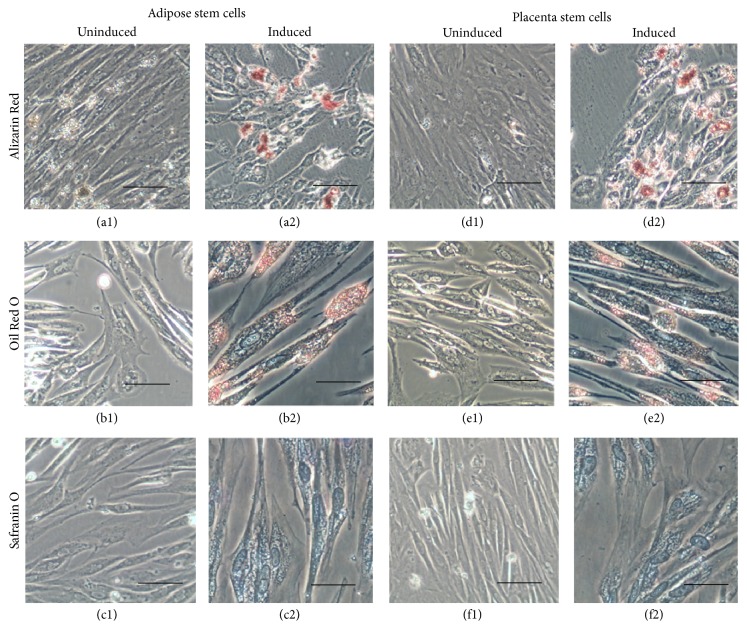
Multipotent differentiation of adipose stem cells and placenta stem cells. After 20 days of induction, the cells were stained with Alizarin Red, Oil Red, and Safranin O. Both kinds of cells could differentiate into osteocytes (a2, d2), adipocytes (b2, e2), and chondrocytes (c2, f2) successfully. And the uninduced groups were cells without induction as negative control (a1–f1). Scale bar = 50 *μ*m.

ASCs and PSCs showed the same morphological features as fibroblast-like adherent cells in passage 1 and passage 3 (Figure 1). In FACS analysis, the two types of cells expressed the same surface proteins, such as CD29, CD34, and CD71. In this study, the expression of CD90 was 80.45% in PSC and 47.1% in ASC (Figure 1). In the multipotency analysis, both types of cells successfully differentiated into chondrocytes, adipocytes, and osteocytes (Figure 2). The experiment showed that these two types of cells have very similar characteristic and function consistent with the characteristics of mesenchymal stem cells.

### 3.2. Characterization of Secretory Proteins

In our MALDI-TOF/TOF analysis, 11 proteins were identified in the CM of both types of cells: vascular endothelial growth factor (VEGF), M-CSF, stromal cell-derived factor-1 (SDF-1), transforming growth factor-*β* (TGF-*β*), tumor necrosis factor-*α* (TNF-*α*), interleukin-1 (IL-1), interleukin-6 (IL-6), hepatocyte growth factor (HGF), insulin-like growth factor-1 (IGF-1), metalloproteinase-2 (MMP-2), and interleukin-8 (IL-8) (Table 1). 11 proteins were exclusively identified in the PSC-CM: monocyte chemotactic protein 1 (MCP-1) (also referred to as chemokine ligand 2, CCL2), SCF, basic fibroblast growth factor (FGF-2), fibroblast growth factor 7 (FGF-7), angiopoietin-1 (ANGP-1), placental growth factor (PGF), adrenomedullin (AM), plasminogen activator (PA), platelet-derived growth factor (PDGF), metalloproteinase-1 (MMP-1), and metalloproteinase-9 (MMP-9) (Table 2). 10 proteins were exclusively identified in ASC-CM: acidic fibroblast growth factor (FGF-1), G-CSF, CM-CSF, pigment epithelium-derived factor (PEDF), metalloproteinase inhibitor 1 (TIMP-1), plasminogen activator inhibitor (PAI), connective tissue growth factor (CTGF), collagen-1, collagen-6, and fibronectin (FN) (Table 3). The details of peptide sequence identification are shown in Supplement 1 of the Supplementary Material available online at http://dx.doi.org/10.1155/2016/7315830.

**Figure 3:**
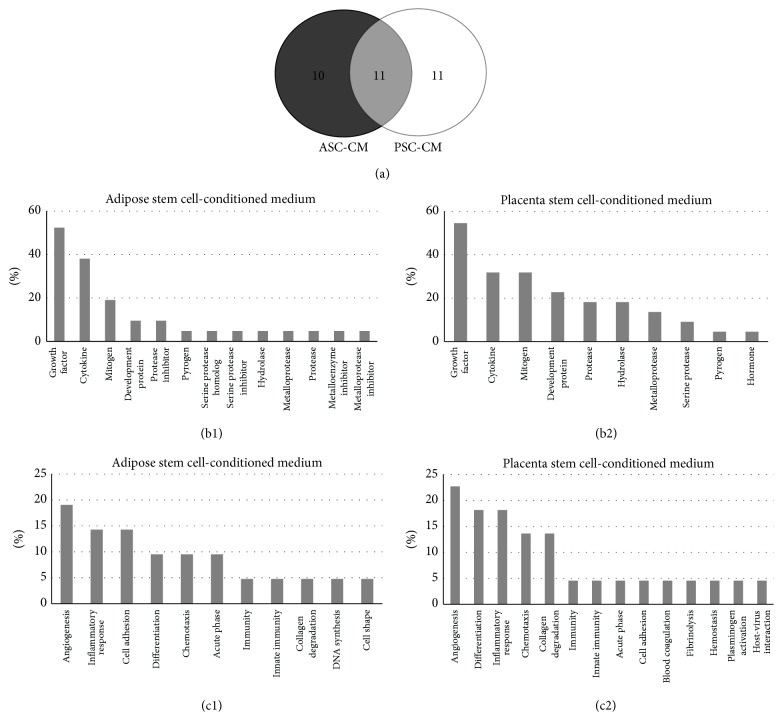
Characteristics of secretory proteins. 11 proteins were identified in both ASC-CM and PSC-CM, 11 proteins were specific in PSC-CM, and 10 proteins were in ASC-CM (a). The molecular function information showed that 52.4% of total protein types of ASC-CM were growth factor (b1); 54.5% of PSC-CM was growth factor (b2). The biological process information showed that 19% of total protein types of ASC-CM had angiogenesis function (c1); 22.7% of total protein types of PSC-CM had angiogenesis function (c2).

According to the molecular function information in the UniProt database, we found that, in the ASC-CM, 52.4% of total protein types were growth factors, 38.1% were cytokines, and 19% were mitogens. In the PSC-CM, 54.5% of total protein types were growth factors, 31.8% were cytokines, 31.8% were mitogens, 22.7% were development proteins, 18.9% were proteases, and 18.9% were hydrolases (Figure 3). Analyzing according to biological processes, we found that in the ASC-CM 19% of total protein types had angiogenesis function, 14.3% were involved in inflammatory response, 14.3% had cell adhesion function, 9.5% had cell differentiation function, 9.5% had chemotaxis function, and 9.5% were involved in acute phase response. In the PSC-CM, 22.7% of total protein types had angiogenesis function, 18.2% were involved in inflammatory response, 18.2% had cell differentiation function, 13.7% had chemotaxis function, and 13.7% had collagen degradation function (Figure 3). Almost all of the examined proteins were secreted proteins; some of these proteins are also found in the extracellular matrix, cell membrane, nucleus, and so forth.

**Figure 4:**
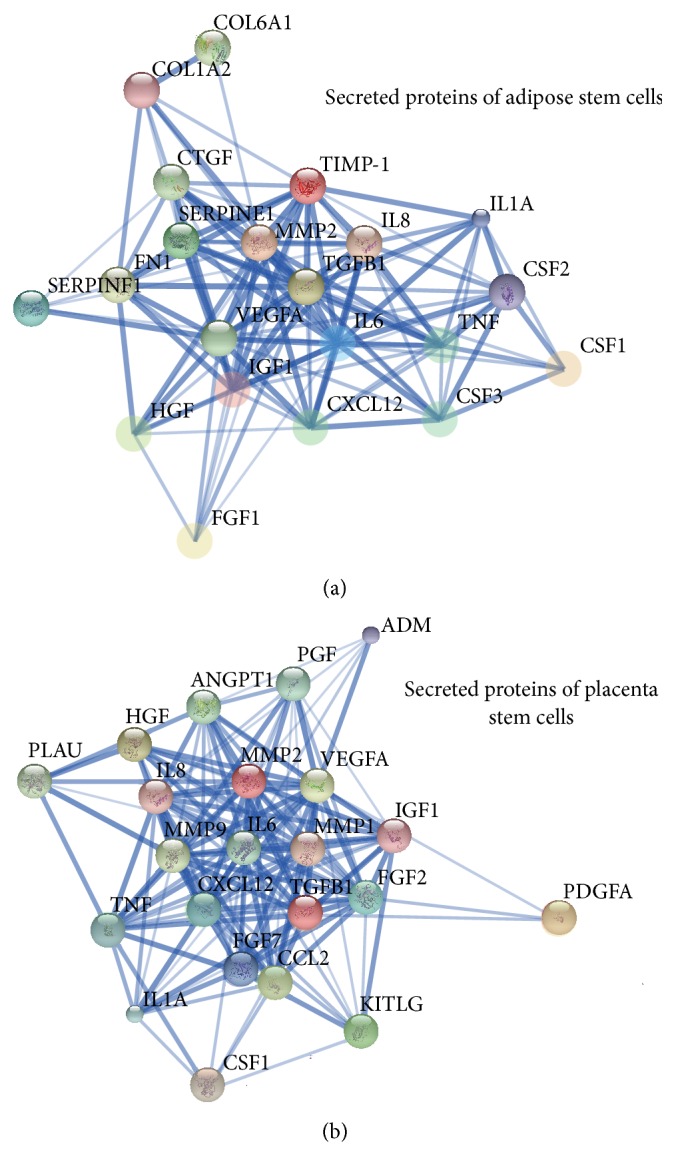
The analysis of protein-protein interaction of secreted proteins of ASC and PSC. The thickness of line represents the strength of evidence. All proteins could interact with others whether in ASC-CM (a) or PSC-CM (b). The relative active proteins in two kinds of CM were IL6, IL8, MMP-2, TGFB1, VEGFA, IGF-1, and CXCL12 (SDF-1). In ASC-CM, the specific active proteins were CTGF, FN, TIMP-1, and SERPINE-1 (PEDF). And in PSC-CM, the specific active proteins were FGF-2, CCL2, FGF-7, MMP-1, and MMP-9.

The protein-protein interactions of secreted proteins of ASC and PSC were analyzed by STRING 10.0. The interaction network was drawn with the thickness of connecting lines representing the strength of evidence (Figure 4). We found that all proteins could interact with others; some proteins had even more active functions for multiple interaction points. The relatively more active proteins in both kinds of CM were IL6, IL8, MMP-2, TGF-*β*, VEGF, IGF-1, and SDF-1 (also known as CXCL12). In the ASC-CM, the unique active proteins were CTGF, FN, TIMP-1, and PEDF (also known as SERPINF1). In the PSC-CM, the unique active proteins were FGF-2, CCL2, FGF-7, MMP-1, and MMP-9. This research shows that most of active proteins in the ASC-CM and the PSC-CM are the same. Comparatively, the ASC-CM had more proteins involved in the function of cell adhesion promotion, metalloproteinase inhibition, and plasminogen activator; the PSC-CM had more proteins involved in the function of cell proliferation promotion, differentiation and migration, inflammatory response, and collagen degeneration.

### 3.3. Antiaging Function in Human Facial Skin

**Figure 5:**
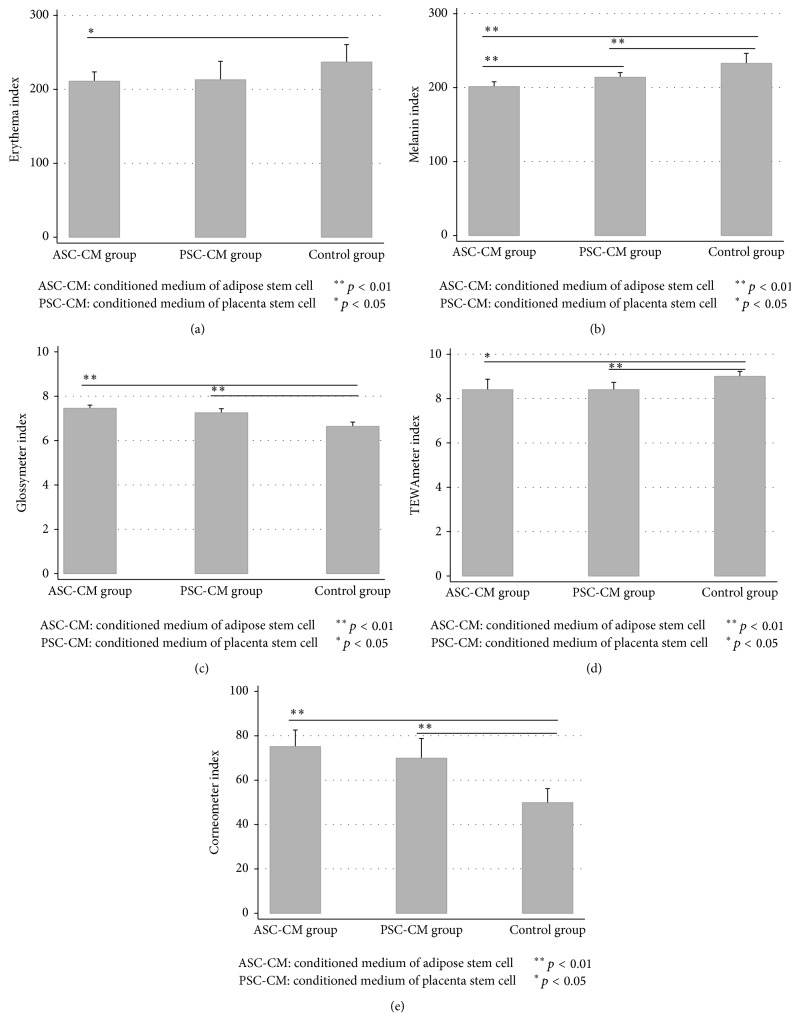
Antiaging functions of ASC-CM and PSC-CM in human facial skin. 15 days after injection, all indexes in ASC-CM group were more improved than those in control group (a–e). All indexes except Erythema in PSC-CM group were more improved than control group (b–e). Only the Melanin index in ASC-CM group was significantly lower than PSC-CM group (b).

There were no significant differences in the facial indexes between the three groups before injection. 15 days after injection, indexes of Erythema, Melanin, Glossymeter, TEWAmeter, and Corneometer of the ASC-CM group showed improvement compared to the control group. Indexes of Melanin, Glossymeter, TEWAmeter, and Corneometer in PSC-CM group also demonstrated improvement compared to the control group. Only the Melanin index of the ASC-CM group was significantly lower than that of the PSC-CM group (Figure 5). According to ANOVA analysis, there were significant differences among three groups in Erythema index (*F* = 10.23, *p* < 0.01), Melanin index (*F* = 47.97, *p* < 0.01), Glossymeter (*F* = 85.13, *p* < 0.01), TEWAmeter (*F* = 13.69, *p* < 0.01), and Corneometer (*F* = 47.42, *p* < 0.01), respectively.

## 4. Discussion

In this study, ASCs and PSCs were used as seed cells because adipose tissue and placenta are ideal stem cell sources in daily medical practice. Furthermore, ASCs and PSCs are also ideal substitutes for autologous bone marrow mesenchymal stem cells due to their similar characteristics. In our study, these two kinds of cells demonstrated similar surface markers and multipotency as other mesenchymal stem cells; these results are the same as those of previous studies.

Utilizing CM is a novel cell-free treatment strategy for clinical applications; concentrated CM has partial function of mesenchymal stem cells [15, 16]. Accumulating evidence indicates that the healing effects of MSCs are mainly related to their unique paracrine properties [17]. In our previous research, ASC-CM was proven to have the ability to promote full-thickness defect skin model healing and human skin laser injury healing [18]. In another research, ASC-CM promoted mouse liver regeneration and increased albumin expression [19]. There has been far less basic and clinical research with PSCs and their CM. In* in vitro* research, the secretory factors of human chorion-derived stem cells are shown to enhance the activation of human fibroblasts and influence wound healing [20].

hPL was used as the serum-free medium for our cell culture because hPL does not induce an immune response when applied* in vivo* and provides adequate nutrition for cell culture [21–23]. In this research, hPL did not affect the expression of cell surface markers or the characteristics of multipotent differentiation. Therefore, hPL is an ideal cell culture medium supplement in serum-free and cell-free research.

In MALDI-TOF/TOF analysis, growth factor proteins accounted for 52.4% and 54.5% of the total secreted proteins in ASC-CM and PSC-CM, respectively. These growth factors can stimulate cell growth, proliferation, differentiation, and maturation [24, 25]. For example, VEGF and FGF promote the formation of artery and vessel [26, 27]. Cytokines, which are important cell signaling molecules, accounted for 38.1% and 31.8% of total secreted proteins in ASC-CM and PSC-CM, respectively. Generally, cytokines are associated with hematopoietic cells and immune system cells; they include chemokine, interferon, interleukin, lymphokine, and tumor necrosis factor [28, 29]. Cytokines are important in modulating the balance between humoral and cell-based immune responses and could be a neutral term to affect cell proliferation [30]. However, some cytokines promote cell division as growth factors, such as G-CSF, M-CSF, and GM-CSF. Mitogen is a chemical substance that stimulates mitosis [31]. Development proteins play an important role in the development of embryos. In addition, many secretory proteins have more than one molecular function. In the analysis of biological processes, we found that proteins involved with angiogenesis and inflammation comprised a major portion in both kinds of CM. However, in the ASC-CM, there were a greater proportion of cell adhesion proteins; in the PSC-CM there was a greater proportion of proteins related to differentiation, chemotaxis, and collagen degradation. This may indicate that ASC-CM is better suited for cell adhesion and that PSC-CM is more appropriate for promoting cell differentiation and chemotaxis.

In the analysis of the protein-protein interaction network, the proteins in the inner layer of the network are considered to have more active protein function. The active proteins in both types of CM included IL6, IL8, MMP2, TGFB1, VEGFA, IGF-1, and CXCL12. Interleukins are a group of cytokines that promotes the development and differentiation of T and B lymphocytes and hematopoietic cells [32]. IL-6 and IL-8 promote hematopoietic stem cell proliferation and differentiation and regulate immune response and chemotaxis [33, 34]. MMP2 is involved in the breakdown of extracellular matrix, such as during embryonic development, reproduction, and tissue remodeling [35]. TGF-B is a multifunctional protein that controls cells proliferation, differentiation, and other functions [36]. VEGFA is active in angiogenesis, vasculogenesis, and endothelial cell growth [37]. IGF has growth-promoting activity [38]. CXCL12 is strongly chemotactic for lymphocytes and mesenchymal stem cells and plays an important role in angiogenesis [39]. The above indicates that the functions of angiogenesis, cell proliferation, differentiation, inflammatory response, and chemotaxis were found in proteins of both kinds of CM.

The unique active proteins in ASC-CM include CTGF, FN, TIMP-1, and SERPINE-1 (PAI). CTGF is a major connective tissue mitoattractant [40]. Fibronectins promote cell motility, adhesion, and increased wound healing [41]. TIMP-1 inhibits metalloproteinase irreversibly by binding to its catalytic zinc cofactor [42]. SERPINE-1 is a type of serine protease inhibitor [43]. Overall, these proteins are more adept at cell adhesion, migration, wound healing, and tissue remodeling.

The unique active proteins in PSC-CM include FGF2, FGF7, CCL2, MMP1, and MMP9. FGF2 and FGF7 play an important role in the regulation of embryonic development, cell survival, cell division, angiogenesis, cell differentiation, and cell migration [44]. CCL2 is a chemotactic factor that attracts monocytes and basophils and augments monocyte antitumor activity [45]. MMP1 and MMP9 cleave types I, II, III, IV, V, VII, and X collagen [46]. The above indicates that proteins unique to PSC-CM are more adept at angiogenesis, cell proliferation, differentiation, cell survival, immunomodulation, and collagen degradation.

Facial aging is a complex process that affects both 3D shape and textures, such as dryness, coarseness, lost luster, and wrinkles. ASCs are most commonly used in plastic surgery as seed cells. ASC-CM has also been proven to play an important role in the prevention of photoaging dermal cells; our previous study has proven that TGF-b is an essential protein in antiaging. In order to compare the effects of ASC-CM and PSC-CM in skin aging, we injected both CM into facial skin and analyzed the facial skin index. After the injection, both CM greatly improved the facial indexes, such as Erythema and Melanin. ASC-CM and PSC-CM were only different in the index of Melanin. Although the secretory proteins of CM of both cell types were detected, the mechanism of antiaging still requires further inquiry.

## Supplementary Material

The details of peptide sequence identification by MALDI-TOF/TOF analysis: The peptide mass fingerprints (PMF) were obtained using the Mascot search engine with a tolerance of 100 ppm and one missed cleavage site. The peptide sequence of fingerprints and matched protein names were showed in this Table. Mr (expt) showed the molecular mass of peptide in experiment; Mr(calc) showed the molecular mass of peptide in calculation. Position indicated the position of detected peptide in protein. 1.1 showed the peptides harvest from placental stem cells' conditioned medium; 1.2 showed the peptides harvest from adipose stem cells' conditioned medium.

## Figures and Tables

**Table 1:** The characterization of secretory proteins in both ASC-CM and PSC-CM.

Protein name	UniProtKB ID	Gene	Molecular function	Biological process	Subcellular location	Family	Function
Vascular endothelial growth factor	P15692	VEGFA	Developmental protein, growth factor, mitogen	Angiogenesis, differentiation	Secreted	PDGF/VEGF growth factor family	Growth factor active in angiogenesis, vasculogenesis, and endothelial cell growth. Induces endothelial cell proliferation, promotes cell migration, inhibits apoptosis, and induces permeabilization of blood vessels

Macrophage colony-stimulating factor	P09603	CSF1	Cytokine, growth factor	Immunity, inflammatory response, and innate immunity	Cell membrane, membrane, secreted	U/A	Cytokine that plays an essential role in the regulation of survival, proliferation, and differentiation of hematopoietic precursor cells, especially mononuclear phagocytes, such as macrophages and monocytes

Stromal cell-derived factor-1	P48061	CXCL12	Cytokine, growth factor	Chemotaxis	Secreted	Chemokine CC family	Chemoattractant active on T-lymphocytes, monocytes, but not neutrophils

Transforming growth factor-*β*	P01137	TGFB1	Growth factor, mitogen	U/A	Extracellular matrix, secreted	TGF-beta family	Multifunctional protein that controls proliferation, differentiation, and other functions in many cell types

Tumor necrosis factor-*α*	P01375	TNF	Cytokine	U/A	Cell membrane, membrane, secreted	Tumor necrosis factor family	It is mainly secreted by macrophages and can induce cell death of certain tumor cell lines

Interleukin-1	P01583	IL1A	Cytokine, mitogen, and pyrogen	Inflammatory response	Secreted	IL-1 family	Produced by activated macrophages; IL-1 stimulates thymocyte proliferation by inducing IL-2 release, B-cell maturation and proliferation, and fibroblast growth factor activity

Interleukin-6	P05231	IL-6	Cytokine, growth factor	Acute phase	Secreted	IL-6 family	Cytokine with a wide variety of biological functions. It is a potent inducer of the acute phase response. Plays an essential role in the final differentiation of B-cells into Ig-secreting cells involved in lymphocyte and monocyte differentiation

Hepatopoietin-A	P14210	HGF	Growth factor, serine protease homolog	U/A	U/A	Peptidase S1 family	Is potent mitogen for mature parenchymal hepatocyte cells, seems to be a hepatotrophic factor, and acts as a growth factor for a broad spectrum of tissues and cell types

Insulin growth factor-1	P05019	IGF1	Growth factor	U/A	Secreted	Insulin family	The insulin-like growth factors, isolated from plasma, are structurally and functionally related to insulin but have a much higher growth-promoting activity

Matrix metalloproteinase-2	P08253	MMP2	Hydrolase, metalloprotease, and protease	Angiogenesis, collagen degradation	Cytoplasm, extracellular matrix, membrane, mitochondrion, nucleus, secreted	Peptidase M10A family	Ubiquitous metalloproteinase that is involved in diverse functions such as remodeling of the vasculature, angiogenesis, tissue repair, tumor invasion, inflammation, and atherosclerotic plaque rupture

Interleukin-8	P10145	CXCL8	Cytokine	Chemotaxis, inflammatory response	Secreted	Chemokine CxC family	IL-8 is a chemotactic factor that attracts neutrophils, basophils, and T-cells, but not monocytes. It is also involved in neutrophil activation

**Table 2:** The characterization of specific secretory proteins in PSC-CM.

Protein name	UniProtKB ID	Gene	Molecular function	Biological process	Subcellular location	Family	Function
Monocyte chemotactic protein 1	P13500	CCL2	Cytokine	Chemotaxis, inflammatory response	Secreted	Chemokine CC family	Chemotactic factor that attracts monocytes and basophils but not neutrophils or eosinophils. Augments monocyte antitumor activity

Kit ligand	P21583	KITLG	Growth factor	Cell adhesion	Cell membrane, cytoplasm, cytoskeleton, and membrane, secreted	SCF family	Ligand for the receptor-type protein-tyrosine kinase KIT. Plays an essential role in the regulation of cell survival and proliferation, hematopoiesis, stem cell maintenance, gametogenesis, mast cell development, migration and function, and melanogenesis

Fibroblast growth factor 2	P09038	FGF2	Developmental protein, growth factor, mitogen	Angiogenesis, differentiation	Nucleus, secreted	Heparin-binding growth factors family	Plays an important role in the regulation of cell survival, cell division, angiogenesis, cell differentiation, and cell migration

Fibroblast growth factor 7	P21781	FGF7	Growth factor, mitogen	U/A	Secreted	Heparin-binding growth factors family	Plays an important role in the regulation of embryonic development, cell proliferation, and cell differentiation

Angiopoietin-1	Q15389	ANGPT1	Developmental protein	Angiogenesis, differentiation	Secreted	U/A	Plays an important role not only in the regulation of angiogenesis, endothelial cell survival, proliferation, migration, adhesion and cell spreading, and reorganization of the actin cytoskeleton, but also in the maintenance of vascular quiescence. Required for normal angiogenesis and heart development during embryogenesis

Placenta growth factor	P49763	PGF	Developmental protein, growth factor, mitogen	Angiogenesis, differentiation	Secreted	PDGF/VEGF growth factor family	Growth factor active in angiogenesis and endothelial cell growth, stimulating their proliferation and migration

Adrenomedullin	P35318	ADM	Hormone	U/A	Secreted	Adrenomedullin family	AM and PAMP are potent hypotensive and vasodilator agents. Numerous actions have been reported, most related to the physiologic control of fluid and electrolyte homeostasis

Plasminogen activator	P00749	PLAU	Hydrolase, protease, and serine protease	Blood coagulation, fibrinolysis, hemostasis, and plasminogen activation	Secreted	Peptidase S1 family	Specifically cleaves the zymogen plasminogen to form the active enzyme plasmin

Platelet-derived growth factor	P04085	PDGFA	Developmental protein, growth factor, mitogen	U/A	Secreted	PDGF/VEGF growth factor family	Growth factor that plays an essential role in the regulation of embryonic development, cell proliferation, cell migration, survival, and chemotaxis

Matrix metalloproteinase-1	P03956	MMP1	Hydrolase, metalloprotease, and protease	Collagen degradation, host-virus interaction	Extracellular matrix, secreted	Peptidase M10A family	Cleaves collagens of types I, II, and III at one site in the helical domain. Also cleaves collagens of types VII and X

Matrix metalloproteinase-9	P14780	MMP9	Hydrolase, metalloprotease, and protease	Collagen degradation	Extracellular matrix, secreted	Peptidase M10A family	Cleaves type IV and type V collagen into large C-terminal three-quarter fragments and shorter N-terminal one-quarter fragments

**Table 3:** The characterization of specific secretory proteins in ASC-CM.

Protein name	UniProtKB ID	Gene	Molecular function	Biological process	Subcellular location	Family	Function
Fibroblast growth factor-1	P05230	FGF1	Developmental protein, growth factor, mitogen	Angiogenesis, differentiation	Cytoplasm, nucleus, secreted	Heparin-binding growth factors family	Plays an important role in the regulation of cell survival, cell division, angiogenesis, cell differentiation, and cell migration

Granulocyte colony-stimulating factor	P09919	CSF3	Cytokine, growth factor	U/A	Secreted	IL-6 family	Granulocyte/macrophage colony-stimulating factors are cytokines that act in hematopoiesis by controlling the production, differentiation, and function of 2 related white cell populations of the blood, the granulocytes, and the monocytes-macrophages

Granulocyte-macrophage colony-stimulating factor	P04141	CSF2	Cytokine, growth factor	U/A	Secreted	GM-CSF family	Cytokine that stimulates the growth and differentiation of hematopoietic precursor cells from various lineages, including granulocytes, macrophages, eosinophils, and erythrocytes

Pigment epithelium-derived factor	P36955	SERPINF1	U/A	U/A	Secreted	Serpin family	Neurotrophic protein; induces extensive neuronal differentiation in retinoblastoma cells

Metalloproteinase inhibitor 1	P01033	TIMP-1	Growth factor, metalloenzyme inhibitor, metalloprotease inhibitor, and protease inhibitor	U/A	Secreted	Protease inhibitor I35 family	Metalloproteinase inhibitor that functions by forming one to one complexes with target metalloproteinases, such as collagenases, and irreversibly inactivates them by binding to their catalytic zinc cofactor

Plasminogen activator inhibitor 1	P05121	SERPINE1	Protease inhibitor, serine protease inhibitor	U/A	Secreted	Serpin family	Serine protease inhibitor

Connective tissue growth factor	P29279	CTGF	U/A	Cell adhesion, DNA synthesis	Extracellular matrix, secreted	CCN family	Major connective tissue mitoattractant secreted by vascular endothelial cells. Promotes proliferation and differentiation of chondrocytes

Collagen alpha-2(I) chain	P08123	COL1A2	U/A	U/A	Extracellular matrix, secreted	Fibrillar collagen family	Type I collagen is a member of group I collagen (fibrillar forming collagen)

Collagen alpha-1(VI) chain	P12109	COL6A1	U/A	Cell adhesion	Extracellular matrix, secreted	Type VI collagen family	Collagen VI acts as a cell-binding protein

Fibronectin	P02751	FN1	U/A	Acute phase, angiogenesis, cell adhesion, and cell shape	Extracellular matrix, secreted	U/A	Fibronectins bind cell surfaces and various compounds including collagen, fibrin, heparin, DNA, and actin. Fibronectins are involved in cell adhesion, cell motility, opsonization, wound healing, and maintenance of cell shape
