# Supplementary material for: The Comparison of Adipose Stem Cell and Placental Stem Cell in Secretion Characteristics and in Facial Antiaging
Source: Stem Cells Int. 2016 Feb 8;2016:7315830. doi: 10.1155/2016/7315830 (PMC4761676; doi:10.1155/2016/7315830)
Supplement: Supplementary file 1 — The details of peptide sequence identification by MALDI-TOF/TOF analysis: The peptide mass fingerprints (PMF) were obtained using the Mascot search engine with a tolerance of 100 ppm and one missed cleavage site. The peptide sequence of fingerprints and matched protein names were showed in this Table. Mr (expt) showed the molecular mass of peptide in experiment; Mr(calc) showed the molecular mass of peptide in calculation. Position indicated the position of detected peptide in protein. 1.1 showed the peptides harvest from placental stem cells' conditioned medium; 1.2 showed the peptides harvest from adipose stem cells' conditioned medium. [file 7315830.f1.pdf]

Supplement 1:

The details of peptide sequence identification by MALDI-TOF/TOF analysis

1.1

**Source:** Placenta stem cells				
**Peptide sequence**	**Protein name**	**Mr (expt)**	**Mr (calc)**	**Position**
**SWSVYVGAR**	VEGF	1023.54	1023.51	162-170
**EICADPK**	MCP-1	774.35	774.36	73-79
**AFLLVQDIMEDTMR**	M-CSF	1680.86	1680.82	85-98
**DNTPNAIAIVQLQELSLR**	M-CSF	1994.02	1994.08	101-118
**TFYETPLQLLEK**	M-CSF	1480.73	1480.78	136-148
**PDCNCLYPK**	M-CSF	1051.42	1051.45	187-195
**STCQSFEPPETPVVK**	M-CSF	1647.71	1647.78	256-270
**DSTIGGSPQPR**	M-CSF	1113.50	1113.54	271-281
**FSNISEGLSNYSIIDK**	SCF	1785.81	1785.88	88-103
**LVNIVDDLVECVK**	SCF	1457.84	1457.78	104-116
**PFMLPPVAASSLR**	SCF	1384.70	1384.75	182-194
**FFESHVAR**	SDF-1	991.51	991.49	34-41

**MVGVGGGDVEDVTPR**	FGF-2	1486.67	1486.71	1-15
**PGGCQISGR**	FGF-2	873.40	873.41	16-24
**GCNGIPGAAAWEAALPR**	FGF-2	1652.85	1652.81	28-44
**WILTWILPTLLYR**	FGF-7	1686.93	1686.99	4-16
**SYDYMEGGDIR**	FGF-7	1304.50	1304.53	55-65
**ESTTDQYNTNALQR**	ANGP-1	1639.71	1639.74	56-69
**DAPHVEPDFSSQK**	ANGP-1	1455.62	1455.66	70-82
**LENYIVENMK**	ANGP-1	1251.59	1251.62	100-109
**LTDVETQVLNQTSR**	ANGP-1	1602.77	1602.82	145-158
**LEIQLLENSLSTYK**	ANGP-1	1649.95	1649.89	159-172
**PSYVELTFSQHVR**	PGF	1561.72	1561.79	115-127
**QSPDMPGDFR**	PGF	1148.54	1148.49	137-146
**EILSILGLPHR**	TGF-β	1246.71	1246.74	57-67
**IPEGEAVTAAEFR**	TGF-β	1388.65	1388.69	163-175
**ISVYQVLQEHLGR**	TGF-β	1540.79	1540.84	192-204
**DVELAEEALPK**	TNF-α	1212.64	1212.62	9-19
**DLSLISPLAQAVR**	TNF-α	1381.75	1381.79	66-78
**QAASVVVAMDK**	IL-1	1117.56	1117.58	63-73
**SLVMSGPYELK**	IL-1	1222.66	1222.63	133-143
**PTLQLESVDPK**	IL-1	1225.63	1225.66	194-204
**YILDGISALR**	IL-6	1119.61	1119.63	59-68
**EALAENNLNLPK**	IL-6	1324.67	1324.70	83-94
**VNTADQCANR**	HGF	1090.46	1090.48	64-73
**QCLWFPFNSMSSGVK**	HGF	1729.86	1729.80	95-109
**EFGHEFDLYENK**	HGF	1526.62	1526.67	111-122
**GLMDHTESGK**	HGF	1073.50	1073.48	221-230
**ISSLPTQLFK**	IGF-1	1132.67	1132.65	4-13
**APQTGIVDECCFR**	IGF-1	1437.68	1437.64	86-98
**MSSSYPTGLADVK**	AM	1354.61	1354.64	48-60
**SPEDSSPDAAR**	AM	1130.46	1130.48	79-89
**TQMIYQQHQSWLR**	PA	1717.78	1717.84	46-58
**VEYCWCNSGR**	PA	1215.46	1215.48	66-75
**ATCYEDQGISYR**	PA	1404.56	1404.60	125-136
**LGLGNHNYCR**	PA	1145.51	1145.54	171-180
**VYTAQNPSAQALGLGK**	PA	1616.81	1616.85	248-263
**FPPHHLTVILGR**	PA	1385.75	1385.79	363-374
**CGWTGPGHSAR**	PDGF	1127.52	1127.49	45-55
**SIEEAVPAVCK**	PDGF	1144.56	1144.58	101-111
**QMQEFFGLK**	MMP-1	1126.53	1126.55	66-74
**WEQTHLTYR**	MMP-1	1232.56	1232.59	109-117
**IENYTPDLPR**	MMP-1	1216.63	1216.61	118-127
**AFQLWSNVTPLTFTK**	MMP-1	1751.87	1751.92	137-151
**VSEGQADIMISFVR**	MMP-1	1550.73	1550.78	152-165

**ELAVQYLNTFYGCPK**	MMP-2	1744.91	1744.85	48-62
**CGNPDVANYNFFPR**	MMP-2	1612.76	1612.71	102-115
**AFQVWSDVTPLR**	MMP-2	1417.69	1417.74	147-158
**IHDGEADIMINFGR**	MMP-2	1586.68	1586.75	162-175
**QSTLVLFPGDLR**	MMP-9	1344.70	1344.74	25-36
**QLAEEYLYR**	MMP-9	1183.56	1183.59	66-76
**EYSTCTSEGR**	MMP-9	1151.75	1151.73	357-366
**FTEGPPLHK**	MMP-9	1131.44	1131.45	425-433
**PQGPFLIADK**	MMP-9	1024.55	1024.53	550-559
**LDSVFEEPLSK**	MMP-9	1262.67	1262.64	567-577
**VIESGPHCANTEIIVK**	IL-8	1708.82	1708.88	54-69

1.2

**Source:** Adipose stem cells				
**Peptide sequence**	**Protein name**	**Mr (expt)**	**Mr (calc)**	**Position**
**MAEGEITTFTALTEK**	FGF-1	1640.85	1640.80	1-15
**LLYCSNGGHFLR**	FGF-1	1378.65	1378.68	28-39
**MAGPATQSPMK**	G-CSF	1117.51	1117.53	1-11
**IQGDGAALQEK**	G-CSF	1128.60	1128.58	54-64
**GPLTMMASHYK**	GM-CSF	1234.54	1234.58	92-102
**DFLLVIPFDCWEPVQE**	GM-CSF	1949.01	1948.93	129-144
**YILDGISALR**	IL-6	1119.64	1119.63	59-68
**EALAENNLNLPK**	IL-6	1324.73	1324.70	83-94
**LAAAVSNFGYDLYR**	PEDF	1558.73	1558.78	54-67
**ELLDTVTAPQK**	PEDF	1213.62	1213.66	124-134
**TSLEDFYLDEER**	PEDF	1515.63	1515.67	226-237
**YGLDSDLSCK**	PEDF	1099.50	1099.49	253-262
**GFQALGDAADIR**	TIMP-1	1232.63	1232.61	71-82
**EPGLCTWQSLR**	TIMP-1	1288.59	1288.62	193-203
**GSYPDAILQAQAADK**	PAI	1546.72	1546.76	88-102
**MVLVNAVYFK**	PAI	1182.63	1182.65	196-205
**LNIGYIEDLK**	PAI	1176,62	1176.64	241-250
**MAEDEVEVYIPQFK**	PAI	1696.75	1696.80	300-313
**MTAASMGPVR**	CTGF	1019.48	1019.49	1-10
**VAFVVLLALCSR**	CTGF	1289.72	1289.75	11-22
**PAVGQNCSGPCR**	CTGF	1187.48	1187.52	23-34
**GLFCHFGSPANR**	CTGF	1304.64	1304.61	79-90
**AGAPGPHGPVGPAGK**	Collagen I	1268.63	1268.66	1-14
**TGHPGTVGPAGIR**	Collagen I	1218.67	1218.65	109-121
**VYCDFSTGETCIR**	Collagen I	1492.69	1492.63	234-246
**EMATQLAFMR**	Collagen I	1196.55	1196.57	290-299
**GDPGEAGPQGDQGR**	Collagen VI	1339.61	1339.58	22-35

**HLFVQDPQTCK**	VEGF	1314.61	1314.64	193-203
**ILNTPNCALQIVAR**	SDF-1	1524.90	1524.84	49-62
**ISVYQVLQEHLGR**	TGF-β	1540.90	1540.84	192-204
**LAGLIGRHGPQNK**	TGF-β	1359.74	1359.77	261-273
**MANVAENSSSDQR**	TGF-β	1407.64	1407.60	315-327
**EALAENNLNLPK**	TGF-β	1324.74	1324.70	83-94
**DGCFQSGFNEETCLVK**	TGF-β	1775.69	1775.75	99-114
**PWCYTLDPHTR**	HGF	1387.42	1387.46	269-279
**GTVNTIWNGIPCQR**	HGF	1557.71	1557.77	315-328
**NYMGNLSQTR**	HGF	1182.53	1182.55	398-407
**HIFWEPDASK**	HGF	1228.57	1228.59	425-434
**SIEEAVPAVCK**	PDGF	1144.60	1144.58	101-111
**CTGCCNTSSVK**	PDGF	1101.41	1101.43	143-153
**WEHGDGYPFDGK**	MMP-2	1406.55	1406.59	176-187
**YGNADGEYCK**	MMP-2	1118.41	1118.43	225-234
**EYNSCTDTGR**	MMP-2	1144.46	1144.45	243-252
**IDAVYEAPQEEK**	MMP-2	1390.61	1390.66	520-531
**DVELAEEALPK**	TNF-α	1212.59	1212.62	9-19
**DLSLISPLAQAVR**	TNF-α	1381.82	1381.79	66-78
**APQTGIVDECCFR**	IGF-1	1437.59	1437.64	86-98
**DSMIWDCTCIGAGR**	FN	1526.67	1526.63	117-130
**CHEGGQSYK**	FN	1007.42	1007.41	141-149
**GNLLQCICTGNGR**	FN	1347.61	1347.64	253-265
**TFYSCTTEGR**	FN	1163.47	1163.49	370-379
**WCGTTQNYDADQK**	FN	1528.59	1528.63	445-457
**GEWTCIAYSQLR**	FN	1425.62	1425.67	504-515
**TYHVGEQWQK**	FN	1274.57	1274.60	2301-2310
**QAASVVVAMDK**	IL-1	1117.60	1117.58	63-73
**SLVMSGPYELK**	IL-1	1222.59	1222.63	133-143
**VIESGPHCANTEIIVK**	IL-8	1708.95	1708.88	54-69
